# Supplementary material for: Causal interplay between lactose intolerance and gut microbiota: a combined bidirectional Mendelian randomization and in vivo validation study
Source: Front Nutr. 2026 Jun 1;13:1803337. doi: 10.3389/fnut.2026.1803337 (PMC13265576; doi:10.3389/fnut.2026.1803337)
Supplement: Supplementary file 1 [file Data_Sheet_1.zip › supplementary materials/Forward/leave one out cross validation/ebi-a-GCST90027539.finngen_R12_E4_LACTONAS.pdf]

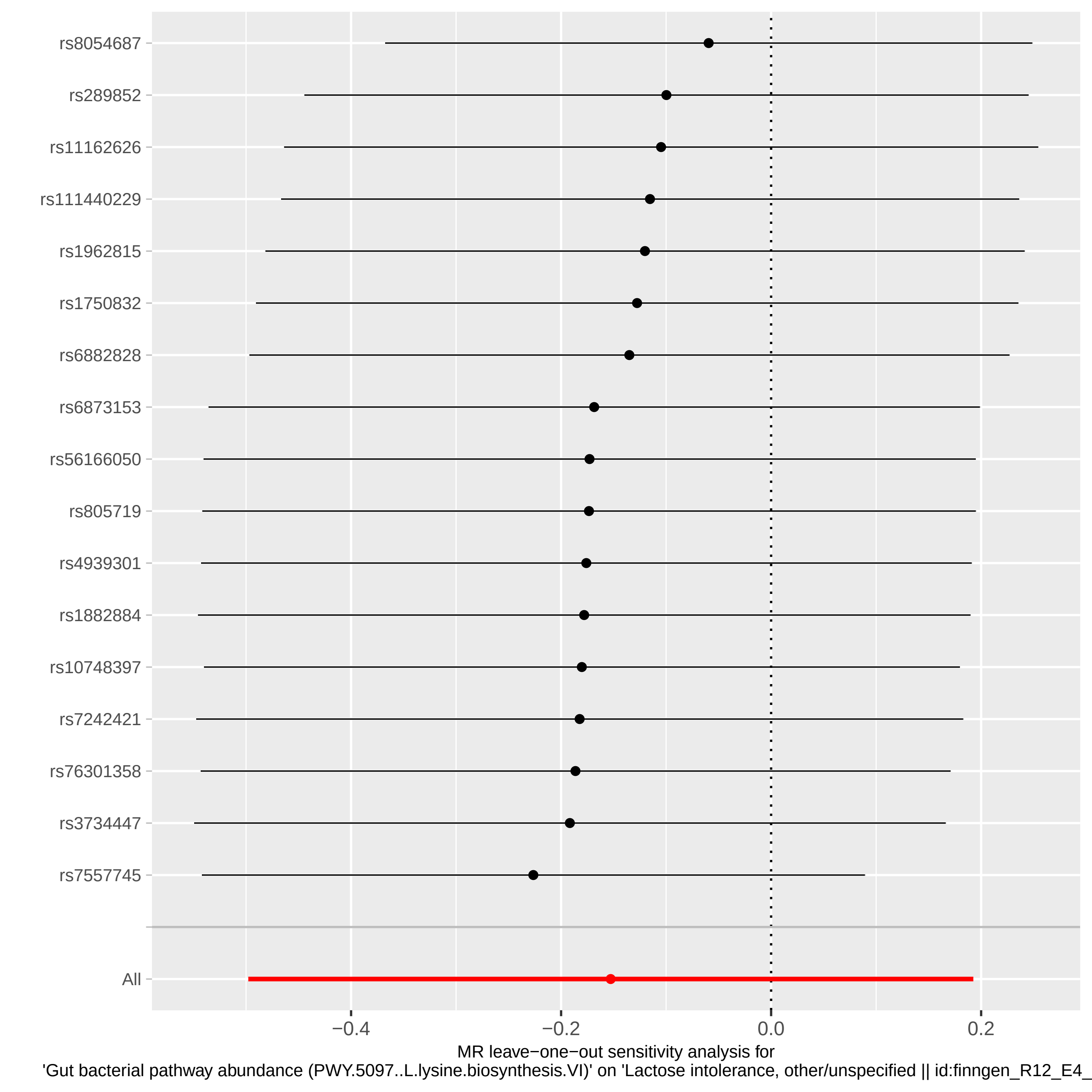

rs8054687
rs289852
rs11162626
rs111440229
rs1962815
rs1750832
rs6882828
rs6873153
rs56166050
rs805719
rs4939301
rs1882884
rs10748397
rs7242421
rs76301358
rs3734447
rs7557745
All
−0.4
−0.2
0.0
0.2
MR leave−one−out sensitivity analysis for
'Gut bacterial pathway abundance (PWY.5097..L.lysine.biosynthesis.VI)' on 'Lactose intolerance, other/unspecified || id:finngen_R12_E4_
